# Presenteeism as a predictor of disability pension: A prospective study among nursing professionals and care assistants in Sweden

**DOI:** 10.1002/1348-9585.12070

**Published:** 2019-07-11

**Authors:** Klas Gustafsson, Gunnar Bergström, Staffan Marklund, Emmanuel Aboagye, Constanze Leineweber

**Affiliations:** ^1^ Department of Clinical Neuroscience, Division of Insurance Medicine Karolinska Institutet Stockholm Sweden; ^2^ Institute of Environmental Medicine, Unit of Intervention and Implementation Research for Worker Health Karolinska Institutet Stockholm Sweden; ^3^ Department of Occupational Health Sciences and Psychology, Faculty of Health and Occupational Studies, Centre for Musculoskeletal Research University of Gävle Gävle Sweden; ^4^ Stress Research Institute, Stockholm University Stockholm Sweden

**Keywords:** disability pension, health care workers, nurses, presenteeism, working conditions

## Abstract

**Objectives:**

The aim of the present study was to examine how presenteeism affects the risk of future disability pension among nursing professionals and care assistants (assistant nurses, hospital ward assistants, home‐based personal care workers, and child care assistants). A specific objective was to compare health and social care employees with all other occupations.

**Methods:**

The study was based on a representative sample of working women and men (n = 43 682) aged 16‐64 years, who had been interviewed between 2001 and 2013 for the Swedish Work Environment Survey conducted every second year since 1989. Information on disability pension was obtained from the Social Insurance Agency's database (2002‐2014). The studied predictors were related to disability pension using Cox's proportional hazard regression with hazard ratios (HR) and 95% confidence interval (CI) and selected confounders were controlled for. The follow‐up period was 6.7 years (SD 4.2).

**Results:**

Health and social care employees with frequent presenteeism showed a particularly elevated risk of future disability pension after adjusting for sex, sociodemographic variables, physical and psychosocial working conditions, and self‐rated health symptoms. In the amalgamated occupational group of nursing professionals and care assistants, the impact on disability pension of having engaged in presenteeism four times or more during the prior year remained significant (HR = 3.72, 95% CI = 2.43‐5.68).

**Conclusions:**

The study suggests that frequent presenteeism contributes to an increased risk of disability pension among nursing professionals and care assistants as well as among all other occupations.

## 1 INTRODUCTION

Sickness presenteeism at work has been approached in several different ways, but according to a review of the research literature on the subject, two main approaches are in use.^1^ The first is to treat it more generally in terms of considering various aspects relevant to the phenomenon of attending work while ill,^2^, ^3^ an approach that dominates research in Europe. The other main approach is to specifically focus on the productivity loss that stems from attending work while ill.^4^ This focus is most common in the occupational medicine literature and among American medical scholars.^5^, ^6^ The present study follows the first approach focus by investigating the association between attending work while ill and subsequent disability pension.

Empirical research on presenteeism has increased dramatically during the past decades.^1^, ^7^ While more than 10 000 scientific publications can be found from the last 5 years using the search term “presenteeism,” a corresponding search among texts from 15 to 20 years ago yields less than 100 publications (Google Scholar Jan 2019, https://scholar.google.se/).

New perspectives and issues have emerged, and a number of significant approaches to understanding the phenomenon of presenteeism have been noted.^1^, ^8^ This is true for research concerned with the causes of presenteeism as well as research that focuses on its effects. The new angles span taking into account a number of aspects, including presenteeism's associations with cultural differences, occupation, gender, age, physical and psychosocial work environments, leadership styles, irreplaceability, collegial support, supervisor support, professional roles, temporary and permanent employment, and work‐family conflict. Studies that focus on consequences indicate that presenteeism may affect health negatively, and that it can be followed by reduced work ability and sickness absence.^1^, ^8^, ^9^, ^10^, ^11^, ^12^, ^13^ Other studies have looked at the risks of presenteeism leading to errors, low productivity, and safety problems at work.^5^, ^14^, ^15^

Some of the earliest studies of presenteeism focused on health care employees such as physicians and nurses.^2^, ^16^, ^17^, ^18^, ^19^, ^20^ Most of these studies focused on factors specifically related to the professional role of health care personnel and on replacement difficulties (ie, impact of perceptions on how easily missed work due to sickness absence could be made up for) that may explain relatively high rates of sickness presence in these occupations. More recently, a systematic review of presenteeism among health care employees has revealed that a wider variety of explanatory factors can contribute to the prevalence of presenteeism, such as psychosocial working conditions, employment conditions, and conditions related to sickness insurance.^21^ According to a Dutch study, there was also an indication that job demands and burnout exhibited a substantial longitudinal relationship with presenteeism among nurses.^22^

Other studies have focused on the associations between presenteeism and financial motives, personal health, consequences for patients' health, low productivity, low quality of work, increased risk of developing health disorders, and risk of contagion for patients.^23^, ^24^

Nurses and care assistants have been found to have an increased risk of presenteeism^2^, ^24^ as well as relatively high sick leave rates, a finding that they have in common with other health care employees.^16^, ^25^, ^26^, ^27^ The most commonly given reason for presenteeism among nurses in Sweden as well as among other health care employees was “not wanting to increase the burden for colleagues” (57% and 52% respectively). A large proportion among health care employees also reported that they could not afford to be sickness absent.^28^

When it comes to the consequences of presenteeism, a range of prospective studies have found increased risks for sickness absence, decreased self‐rated health, musculoskeletal disorders, depression, depersonalization, and exhaustion in a range of occupational groups.^8^, ^9^, ^22^, ^29^, ^30^ In a couple of prospective studies, Bergstrom and collaborators found an increased risk for long‐term sickness absence 2 years after reporting presenteeism among different working populations.^12^, ^13^, ^31^ In one of the few prospective studies on health consequences of presenteeism among health care personnel, Dellve and collaborators reported an association between presenteeism and both poor health and sickness absence in a 2‐year follow‐up study of health care personnel.^32^ To the best of our knowledge, no prospective study has attempted to estimate the risk for disability pension, which is a common outcome of long‐term sickness absence among health care employees after frequent periods of presenteeism.

The aim of the present study was to investigate how presenteeism affects the future risk for disability pension among nursing professionals and health and social care assistants. Specifically, we wanted to compare health and social care employees with a wide range of other occupations to detect similarities or differences.

## 2 METHODS

### 2.1 Study design and participants

The primary data sources were the Swedish Work Environment Surveys (SWES) and two population registers. Developed by Statistics Sweden (SCB) on behalf of the Swedish Work Environment Authority, the SWES has been conducted every second year since 1989. The surveys cover specific years and were administered to random samples of the Swedish employed population aged 16‐64 years through telephone interviews and a supplementary postal questionnaire by Statistics Sweden. An official translation of the survey questionnaire into English is available at Statistics Sweden (www.scb.se). In the present study, data from 46 675 individuals from seven iterations of the surveys between 2001 and 2013 were included. The data cover a broad range of physical and psychosocial working conditions.^28^ The response rate for 2001‐2013 varied between 77% and 66%.

Information on granted disability pensions for the period 2002‐2014 was attained from the Micro Data for Analysis of Social Insurance database at the Swedish Social Insurance Agency. In Sweden, a disability pension can be granted to persons aged 19‐64 years who have been medically assessed to be unable to work due to reduced health into the foreseeable future. It can be granted for 100%, 75%, 50% or 25% of an employee's regular working hours and covers on average about 60% of the income loss.^33^ Data on background factors for this study (2001‐2013) were derived from The Longitudinal Database for Health Insurance and Labor Market Studies (LISA) at Statistics Sweden.

Men and women who had obtained a disability pension prior to being interviewed or during the year of the interview (n = 2993) were excluded. Of the 43 682 remaining individuals, 992 (2.27%) were granted disability pension within the 2002‐2014 follow‐up period. Table 1 shows the characteristics of the study group.

**Table 1:** Description of the study group according to sex, age, occupation at time of interview (2001‐2013), and disability pension status, 2002‐2014 (n = 43 682)

	No disability pension		Disability pension after interview	
	n	%	n	%
Answering questionnaires 2001‐2013	42690	97.7	992	2.3
Sex				
Men	19378	98.5	287	1.5
Women	23312	97.1	705	2.9
Age at interview (years)				
16‐29	5758	99.7	16	0.3
30‐39	9756	99.1	92	0.9
40‐49	11466	97.9	244	2.1
50‐64	15710	96.1	640	3.9
Occupation at interview				
Nursing professionals	1617	97.1	48	2.9
Care assistants	5725	96.0	240	4.0
All other occupations	35348	98.1	704	2.0

#### 2.1.1 Occupation (stratification variable)

The occupational category of interest in this study consists of employees in health and social care, grouped according to the 1996 Swedish Standard Classification of Occupations (SSYK96) (https://www.scb.se/). It contains two subgroups. The first subgroup, “Nursing professionals,” (n = 1665) includes nursing and midwifery professionals and nursing associate professionals (SSYK96, 223 and SSYK96, 323) with a university degree and who were working in hospitals or other health care organizations. The second subgroup, “Care assistants,” (n = 5965) consists of employees in personal care and related workers such as assistant nurses (SSYK96, 513), also including hospital ward assistants, home‐based personal care workers, and childcare assistants. The educational requirement for these occupations is generally upper‐secondary education. For certain analyses, these two categories were merged into one category. The comparison group was comprised of all other occupations in the SWES (n = 36 052).

### 2.2 Measurements

#### 2.2.1 Outcome variable

All cases of granted disability pension during 2002 to 2014, regardless of the diagnostic category, were used (n = 992).^33^ No distinction was made concerning full‐time or part‐time disability pension in the analyses.

#### 2.2.2 Exposure variables

Data related to presenteeism were obtained from the Swedish Work Environment Survey (SWES, 2001‐2013).^28^

The following item was chosen as the indicator for *presenteeism* 2, 22, 34: “How many times during the past 12 months have you worked, even though you really should have not worked given your medical condition?” The 4‐point response scale was: Never (1), once (2), two to three times (3), four times or more (4).

#### 2.2.3 Potential confounders

*Sex, age at time of interview* (divided into 16‐29, 30‐39, 40‐49, and 50‐64 years), *country of birth* (born in Sweden or other country), and *sector of employment* (public sector or private sector) were selected as potential confounders. This information was obtained from the LISA database.

Data on physical and psychosocial working conditions were obtained from the SWES 2001‐2013. Several methods that tested reliability and validity of each single item were used when the survey in SWES was constructed. One method was based on testing the items at work places, where the actual conditions were well known. Further, a number of validation studies were conducted, where responses to different types of items were compared.^28^ The following item was chosen as the indicator of *physically strenuous work postures:* “Do you bend or twist yourself in your work in the same way repeatedly in an hour, for several hours during the same day?” The 5‐point response scale (every day, 1 day of 2, 1 day of 5, 1 day of 10, not at all) was dichotomized closest to the upper quartile to indicate the most adverse conditions. The dichotomized response alternatives are No (≤1 day of 2) and Yes (every day).

*Psychosocial working conditions* included job demands, job control, and support from supervisors.

The following item was chosen as an indicator of *job demands*: ‘‘Do you have so much work that you miss lunch, work late, or take work home? The 5‐point response scale (every day, 1 day of 2, 1 day of 5, 1 day of 10, not at all) was dichotomized closest to the upper quartile to indicate the most adverse conditions. The dichotomized response alternatives are Yes (≥1 day of 2) and No (≤1 day of 5).

The following item was chosen as an indicator of *job control*: ‘‘Do you have the opportunity to determine your work pace?’’ The 6‐point response scale (nearly all the time, about 3/4 of the time, 1/2 of the time, about 1/4 of the time, about 1/10 of the time, no, not at all) was dichotomized closest to the upper quartile to indicate the most adverse conditions. The dichotomized response alternatives are No (≤1/10 of the time) and Yes (≥1/4 of the time).

One item covered *support from supervisors*: “Are you able to get support and encouragement from supervisors when work feels difficult?” Responses were given on a 4‐point scale (always, mostly, mostly not, never) and dichotomized into Yes (always, mostly) and No (mostly not, never).

Since poor health could contribute to presenteeism and is a prerequisite for being granted disability pension, individuals' health symptoms were controlled for. The data on *self‐reported health symptoms* were obtained from the SWES 2001‐2013. Among the numerous available indicators of poor health in the survey, the three most commonly reported were selected.

The following three items were chosen as indicators of *health symptoms:*

“Have you experienced pain in your upper back or neck after work during the past three months?”

“Have you had trouble sleeping during the last three months?”

“Have you felt tired and listless during the last three months?”

The 5‐point response scale (every day, 1 day of 2, 1 day of 5, 1 day of 10, not at all) was dichotomized closest to the upper quartile to indicate the most adverse conditions. The dichotomized response alternatives are No (≤1 day of 5) and Yes (≥1 day of 2).

*Long‐term sickness absence*, at the time of the interviews (spanning 2001‐2013) was defined according to the number of ongoing periods of medically certified sickness absence lasting 60 days or more, as recorded in the Swedish Social Insurance registers and obtained from the LISA database.^35^ The categories are No (<60 days) and Yes (≥60 days). This item was analyzed separately. However, due to the fact that almost all individuals with disability pension had been on long‐term sickness absence, long‐term sickness absence was not included as a confounder in the main analyses.

### 2.3 Statistical analyses

The selected participants from the SWES surveys were consecutively added to the cohort, and the follow‐up period for each sub‐cohort started the year after the interview (1 January 20011 January 2013). The follow‐up period for the participants ended the year they reached 64 years, when they went on disability pension, emigrated or died or on December 31, 2014 (the final cutoff date), whichever came first. The mean number of years of follow‐up was 6.7 years (SD 4.2). Hazard ratios (HRs) of being granted a disability pension, with 95% confidence intervals (CI), were estimated using Cox's proportional hazards regression analysis. All statistical analyses were conducted with SAS, version 9.4, statistical software (SAS Institute, Inc, Cary, North Carolina) using the PHREG procedure.

The statistical analyses were conducted in two phases. In the first phase, presenteeism, occupation, sociodemographic characteristics (sex, age as a categorical variable, country of birth), sector of employment, working conditions (strenuous work postures, job demands, job control, support from supervisors), and health symptoms were analyzed, adjusting for age as a continuous variable and year of interview (Model 1). Next, adjustments were made for all variables except health symptoms (Model 2). In the final model, health symptoms were also added to the regression analyses (Model 3).

In the second phase, the associations between presenteeism, sociodemographic characteristics, sector of employment, working conditions, and health symptoms were analyzed and stratified by occupation into the categories of nursing professionals, care assistants, and all other occupations. A possible interaction effect of the combination of presenteeism and occupation was also tested.

## 3 RESULTS

More women than men were granted disability pension in the follow‐up period, and the proportion of individuals who were granted disability pension increased with age. During the follow‐up time, 2.9% of all nursing professionals received a disability pension, compared to 2.0% among the “all other occupations” group. The highest proportion was among care assistants (4.2%).

Table 2 presents the HRs for future disability pension among all occupations according to presenteeism, sociodemographic variables, working conditions, and health symptoms. After adjusting for age and year of interview (Model 1), the HRs for risk of disability pension increased among individuals who frequently engaged in presenteeism (ie, 4 times or more). Further, these adjustments also led to increased HRs among care assistants, older persons, foreign‐born individuals, and individuals in public employment. This was also the case among individuals reporting strenuous work postures, low job control, or low support from their supervisor as well among those reporting health symptoms such as upper back or neck pain, being tired and listless, and having sleeping problems. No significant effects of sex or high job demands emerged. Further, no significant interaction effect was found for presenteeism and occupation (1.14, 95% CI 0.94‐1.38; *P* value = 0.1909). This means that there was no difference between the occupations in the association between presenteeism and disability pension.

**Table 2:** Presenteeism, occupation, sociodemographic variables (sex, age, country of birth*),* sector of employment, working condition and health factors, and risk of disability pension, 2002‐2014, interviewed 2001‐2013

	Disability pension, all occupations^a^ (n = 43 682, (992 cases))										
	Prevalence		Model 1			Model 2			Model 3		
	P^b^	n^c^	HR^d^	CI		HR^e^	CI		HR^f^	CI	
Presenteeism											
Never	30.1	191	1			1			1		
Once	21.1	126	1.16^g^	0.93	1.45	1.13^i^	0.89	1.42	1.09^j^	0.86	1.39
2 to 3 times	32.2	292	**1.68** h	1.40	2.01	**1.55** h	1.28	1.87	**1.32** k	1.08	1.61
4 times or more	16.6	365	**4.17** h	3.49	4.97	**3.87** h	3.21	4.68	**2.81** h	2.28	3.46
Occupation											
All other occupations	82.5	704	1			1			1		
Nursing professionals	3.8	48	1.17	0.87	1.56	0.88	0.64	1.20	0.90	0.65	1.24
Care assistants	13.7	240	**1.76**	1.52	2.03	**1.18**	1.00	1.40	1.17	0.98	1.40
Sex											
Men	45.0	287	1			1			1		
Women	55.0	705	1.56	0.38	6.47	**1.44**	1.23	1.69	**1.26**	1.07	1.49
Age at interview (years)											
16‐29	13.2	16	1			1			1		
30‐39	22.5	92	**2.60**	1.49	4.53	**2.18**	1.24	3.83	**2.18**	1.24	3.83
40‐49	26.8	244	**4.63**	2.52	8.48	**3.43**	1.84	6.36	**3.21**	1.71	6.01
50‐64	37.4	640	**6.36**	3.07	13.18	**4.48**	2.11	9.49	**4.22**	1.96	9.07
Country of birth											
Sweden	92.1	896	1			1			1		
Other country	7.9	96	**1.36**	1.10	1.68	1.07	0.85	1.34	0.96	0.76	1.22
Sector of employment											
Private	53.6	302	1			1			1		
Public organization	46.4	672	**1.65**	1.44	1.90	**1.39**	1.18	1.62	**1.33**	1.13	1.57
Strenuous work postures											
Bent or twist repeatedly											
No (≤1 d of 2)	78.1	630	**1**			1			1		
Yes (Every day)	21.9	342	**1.60**	1.40	1.83	**1.32**	1.15	1.52	1.15	0.99	1.33
Job demands											
No (<1 d of 5)	79.8	763	1			1			1		
Yes (≥1 d of 2)	20.2	210	1.12	0.96	1.30	**0.80**	0.68	0.94	**0.70**	0.59	0.84
Job control											
Yes ( ≥1/4 of time)	76.0	648	1			1			1		
No (≤1/10 of time)	24.0	333	**1.69**	1.48	1.93	**1.26**	1.09	1.46	**1.20**	1.04	1.40
Support from supervisors											
Always—mostly	65.7	566	1			1			1		
Mostly not—never	34.3	411	**1.24**	1.09	1.41	1.12	0.98	1.28	1.03	0.89	1.18
Health symptoms											
Upper back or neck pain											
≤1 d of 5	76.3	505	1						1		
>1 d of 2	23.7	424	**2.58**	2.27	2.94				**1.60**	1.38	1.86
Tired and listless											
≤1 d of 5	79.7	570	**1**						1		
>1 d of 2	20.3	393	**2.82**	2.48	3.20				**1.77**	1.51	2.07
Sleeping troubles											
≤1 d of 10	78.8	619	**1**						1		
≥1 d of 5	21.2	353	**1.86**	1.63	2.12				1.05	0.90	1.23

Bold = statistical significant at the *P* < 0.05 level. Bold HR = statistical significant at the *P* < 0.05 level.

a All incident cases of disability pension (n = 992).

b Prevalence (P) of the exposure category (%).

c Number of cases (n).

d Model 1. Hazard ratio (HR) of disability pension with 95% confidence interval (CI), adjusted for age (continuous variable) and year of interview.

e Model 2. Hazard ratio (HR) of disability pension with 95% confidence interval (CI) for adjusted for age (continuous variable), year of interview, and all other exposures representing sociodemographic characteristics and working conditions.

f Model 3. Hazard ratio (HR) of disability pension with 95% confidence interval (CI), adjusted for age (continuous variable), year of interview, and all other exposures representing sociodemographic characteristics and working conditions, including health symptoms.

g *P*‐value = 0.2014.

h *P*‐value < 0.0001.

i *P*‐value = 0.3187.

j *P*‐value = 0.4714.

k *P*‐value = 0.0076.

When model 1 was extended to include control for occupation, sociodemographic variables, sector of employment, and working conditions—the HRs that reported repeated work presenteeism were found to be high (Table 2, Model 2). The HRs for age, sector of employment, strenuous work posture, and low job control also remained significant. Thus, except for country of birth and support from superior, it was found that most variables had an independent association with future disability pension, although most estimates were slightly reduced. Significant effects of sex and job demands on future disability pension emerged in this model.

In the final model, in which health symptoms were additionally controlled for, presenteeism was still a significant predictor of future risk of disability pension. The same was true for women, all age groups 30 years and older, public employment, low job control, reported upper back or neck pain, and feeling tired and listless (Table 2, Model 3). However, in this model there were no significant differences between the occupational groups.

### 3.1 Stratified analyses

The importance of presenteeism, sociodemographic characteristics, working conditions, and health symptoms were analyzed and stratified by occupation. Stratified analyses were used due to the fact that health care and care work are in many ways different from other occupations.

For the amalgamated occupational group of nursing professionals and care assistants, the impact of presenteeism on future disability pension was strong (Table 3) after controlling for age at interview and year of interview. Being in an age group of 30 years old or older, being exposed to strenuous work postures, and reporting health symptoms were associated with elevated risks of disability pension among health care and care employees. Even after adjustment for sociodemographic characteristics, working conditions, and health symptoms, the risk estimates for disability pension were still high for both frequent and less frequent presenteeism. A high risk was particularly the case among employees over the age of 40 in health and social care. No significant effects of differences in gender, employment sector, country of birth, or working conditions on future disability pension were found among these groups.

**Table 3:** Presenteeism, sociodemographic variables (sex, age, country of birth*),* sector of employment, working conditions, and risk of disability pension, 2002‐2014, interviewed 2001‐2013

	All other occupations n = 36052, (704 cases)						Nursing professionals and care assistants n = 1665 + 5965 = 7630, (288 cases)					
	P^a^	n^b^	HR^c^	HR^d^	CI	CI	P^a^	n^b^	HR^c^	HR^d^	CI	CI
Presenteeism												
Never	30.6	148	1	1			27.8	43	1	1		
Once	21.2	84	1.02 e	0.97^g^	0.73	1.29	20.7	42	**1.55** i	1.50^k^	0.94	2.38
2 to 3 times	31.9	213	**1.62** f	1.24^h^	0.98	1.56	33.8	79	**1.82** j	**1.59** l	1.05	2.42
4 times or more	16.3	248	**3.82** f	**2.56** f	2.01	3.25	17.6	117	**5.02** f	**3.72** f	2.43	5.68
Sex												
Men	52.5	271	1	1			9.8	16	1	1		
Women	47.5	433	**1.61**	**1.27**	1.07	1.51	90.3	272	1.23	1.21	0.68	2.13
Age at interview (years)												
16‐29	12.9	13	1	1			14.7	3	1	1		
30‐39	23.1	63	**2.17**	**1.90**	1.01	3.59	20.0	29	**4.31**	3.16	0.90	11.13
40‐49	26.9	167	**3.99**	**2.86**	1.41	5.80	26.6	77	**6.75**	**4.38**	1.11	17.24
50‐64	37.1	461	**5.86**	**4.23**	1.77	10.12	38.6	179	**7.64**	4.21	0.83	21.26
Country of birth												
Sweden	92.5	639	1	1			90.4	257	1	1		
Other country	7.5	65	**1.32**	0.99	0.75	1.31	9.6	31	1.44	0.91	0.58	1.43
Sector of employment												
Private	62.2	287	1	1			12.6	15	1	1		
Public organization	37.8	407	**1.51**	**1.36**	1.14	1.61	87.4	265	1.46	1.25	0.72	2.15
Strenuous work postures												
Bent or twist repeatedly										s		
No (≤1 d of 2)	78.7	445	1	1			75.0	185	1	1		
Yes (Every day)	21.3	244	**1.68**	**1.20**	1.01	1.43	25.0	98	**1.38**	1.07	0.81	1.41
Job demands												
No (<1 d of 5)	78.8	533	1	1			84.5	230	1	1		
Yes (≥1 d of 2)	21.2	158	1.11	**0.66**	0.54	0.81	15.5	52	1.31	0.81	0.57	1.15
Job control												
Yes (≥1/4 of time)	79.2	484	1	1			60.9	164	1	1		
No (≤1/10 of time)	20.8	212	**1.83**	**1.30**	1.09	1.56	39.1	121	1.14	1.03	0.80	1.34
Support from supervisors												
Always—mostly	64.5	379	1	1			66.9	187	1	1		
Mostly not—never	34.5	312	**1.36**	1.10	0.93	1.30	33.1	99	1.02	0.84	0.64	1.11
Health symptoms												
Upper back or neck pain												
≤1 d of 5	77.4	372	1	1			71.4	133	1	1		
>1 d of 2	23.6	290	**2.58**	**1.59**	1.33	1.90	28.7	134	**2.38**	**1.62**	1.23	2.14
Tired and listless												
≤1 d of 5	80.5	405	1	1			75.7	165	1	1		
>1 d of 2	19.5	278	**2.95**	**1.81**	1.50	2.18	24.3	115	**2.38**	**1.63**	1.21	2.19
Sleeping troubles												
≤1 d of 10	78.8	434	1	1			79.0	185	1	1		
≥1 d of 5	21.3	25	**1.89**	1.06	0.88	1.28	21.0	98	**1.81**	1.04	0.77	1.41

Bold HR = statistical significant at the *P* < 0.05 level.

The analyses are stratified and based on occupation using univariate and multivariate analyses. Further, all incident cases of disability pension, including unspecified diagnoses (n = 992).

a Prevalence (P) of the exposure categories (%).

b Number of cases (n).

c Hazard ratio (HR), and 95% confidence interval (CI), adjusted for age at interview (one‐year intervals), year of interview.

d Hazard ratio (HR), and 95% confidence interval (CI), adjusted for all other exposure representing sociodemographic characteristics and working conditions, including health symptoms.

e *P*‐value = 0.8781.

f *P*‐value < 0.0001.

g *P*‐value = 0.8309.

h *P*‐value = 0. 0.0706.

i *P*‐value = 0.0446.

j *P*‐value = 0.0016.

k *P*‐value = 0.0899.

l *P*‐value = 0.0301.

A similar pattern of associations between presenteeism and disability pension was found among “all other occupations”. Frequent presenteeism resulted in significantly elevated HRs even in the model where all confounders were controlled for. Among “all other occupations”, being a woman, aged above 30, being employed in the public sector, having low job control, and having strenuous work postures, reporting health symptoms all increased the risk for future disability pension. No significant effects of country of birth or support from supervisors were found. Contrary to what one might expect, high job demands were associated with a decreased risk of future disability pension in the “all other occupations” group.

Additional analyses with “all other occupations,” where long‐term sickness absence was included as a potential confounder, were conducted. This did not change the main findings, but the HRs for future disability pension among those with frequent presenteeism (four times or more) were reduced by about 25% in the final model that included all confounders and long‐term sickness absence (HR 2.04 95% CI 1.66‐2.51).

## 4 DISCUSSION

The aim of the present study was to analyze the relations between the risk of future disability pension and presenteeism, sociodemographic factors, working conditions, and health symptoms among nursing professionals and care assistants. All other occupations (in the SWES) constituted the reference group.

The study findings suggest that presenteeism was a strong predictor of future disability pension among nursing professionals and care assistants. Among the confounders, age was the strongest predictor of disability pension in the group of nursing professionals and care assistants, most pronounced among individuals aged 50‐64 years. This indicated that old age was an independent predictor of disability pension in these occupations.

The finding that presenteeism was related to a high risk for future disability pension among health care and care employees is in line with results from a previous prospective study among employees in public health care which showed that sickness attendance increased the risk of long‐term sick leave and poor work ability.^32^ This indicates, as expected, not only that being granted disability pension is strongly related to sickness absence, but it also shows that frequent presenteeism may further elevate the risk of disability pension. Similar results have been reported for other occupational groups.^12^, ^13^, ^34^ However, our finding of high HRs for disability pension related to presenteeism may be somewhat surprising considering that health symptoms were included in the rigorous adjustment for potential confounders.

This may be interpreted in several ways. For one, frequent presenteeism may be more common among some individuals with deteriorating health because they regard the personal financial cost of frequently engaging in sickness absence as too high. In Sweden, the first day of sickness absence is not compensated for and the income loss for the following days is about 20% of the person's regular earnings. Another interpretation is that the impetus for presenteeism may stem from the professional ideals or pressure to work in these occupations, which may increase their threshold for reporting sick—thus increasing the proportions of employees attending work with more serious health problems. This interpretation is in agreement with qualitative and quantitative studies of self‐reported reasons for presenteeism, which indicated that health and social care employees share strong ideals on work ethics and health.^36^, ^37^, ^38^

Many other studies have found aspects such as perceived irreplaceability, professional norms, workplace cultures, negative effects on patients, and economic restrictions on taking sick leave to underlie employees' reasons for presenteeism.^17^, ^20^, ^25^, ^36^, ^37^, ^38^, ^39^ In this study, it was not possible to examine the reasons behind presenteeism. Further research is needed to elucidate the reasons for sickness absence among health and social care employees.

As presenteeism entails reduced health, it is difficult to separate well‐known negative effects of reduced health from the effects of presenteeism itself. This problem exists in presenteeism studies that focus on health as well as those that focus on productivity. In the present study, even when health at baseline was taken into account, presenteeism remained as an independent predictor of future disability pension. An additional separate analysis, where long‐term sickness absence was added as a confounder in the full model, showed, as expected, a reduced risk of disability pension related to presenteeism. Nevertheless, presenteeism remained an independent predictor of disability pension. The assumption that individuals with high rates of presenteeism have low rates of sickness absence is not generally true and therefore methods that include an examination of factors that could affect both sickness absence and presenteeism should be applied.^40^

### 4.1 Strengths and limitations

The present study has several strengths: the prospective design, the population‐based sample, and the use of registry data to complement data from personal interviews. The number of interviews was large and based on representative samples with satisfactory response rates. Information on disability pension was obtained from high‐quality national registers. Since poor health could contribute to presenteeism and is a prerequisite for being granted disability pension, individuals' health symptoms were controlled for.

The present study also has limitations. There was no information available on changes that might have occurred in the work environments during the follow‐up time. Also, the fact that all exposures and confounders were measured at least 1 year ahead of the outcome was likely to have reduced some of the problems related to causal interference. Another potential limitation was that self‐reported health symptoms were assessed at the same point in time as presenteeism and working conditions. This may have affected individual responses in that those who had health problems may have been more likely to notice the negative aspects of their physical or psychosocial working conditions—and those who reported frequent presenteeism may have been more likely to report poor health.

## 5 CONCLUSIONS

The study suggests that presenteeism is an independent predictor of future disability pension among nursing professionals and care assistants, even after adjusting for self‐rated health symptoms. Among the confounders, age was the strongest predictor of disability pension. These results may indicate that frequent presenteeism among health care employees is an early indicator for future disability pension. When it comes to implementing preventative workplaces measures, making improvements to physical and psychosocial working conditions that are designed to reduce health problems may prove effective for reducing presenteeism.

## DISCLOSURE

*Approval of the research protocol*: Data are registered information that originate from Statistics Sweden (SCB) and the Social Insurance Agency (SIA). The compilation of the data set was conducted by Statistics Sweden and given to research team in anonymized format. *Informed consent*: The data from Statistics Sweden are based on informed consent to answer the SWES between 1993 and 2013. Data from the SIA were information about granted disability pensions and were collected for research purposes without consent from the individual. The Swedish law on Research Ethics states that research use of register data that have been given without consent and contain sensitive information, such as health conditions, must get approval from a Regional Research Ethics committee. Such an approval must be sought for research use of personal information even where anonymization has taken place after the linkage occurs. The research protocol was approved by the Regional Research Ethics Committee in 2015 Stockholm, Sweden (Dnr: 2015/2203‐31/5 and Dnr: 2018/5:2). Other researchers may obtain the same data in the same manner as we got it from Statistics Sweden, URL: http://www.scb.se/.* Registry and the registration no. of the study*: Trial No. K824435003 (A), Karolinska institutet. *Animal studies*: This is not an animal study. *Conflict of interest*: The authors have no conflicts of interest to declare.

## AUTHOR CONTRIBUTIONS

All authors met the criteria for authorship, and have approved the final article. Klas Gustafsson: Contributed to conception, design, data analysis, interpretation of data, and writing the article. Staffan Marklund: Contributed to conception, design, interpretation of data, and critical revision of the article for intellectual content. Gunnar Bergström: Contributed to conception, design, interpretation of data, and critical revision of the article for intellectual content. Constanze Leineweber: Contributed to acquisition of data, conception, design, interpretation of data, and critical revision of the article for intellectual content. Emmanuel Aboagye: Contributed to design, conception, interpretation of data, and critical revision of the article for intellectual content.

## References

[1] Johns G . Presenteeism in the workplace: a review and research agenda. J Org Behav. 2010;31(31):519‐542.

[2] Aronsson G , Gustafsson K , Dallner M . Sick but yet at work. an empirical study of sickness presenteeism. J Epidemiol Community Health. 2000;54(7):502‐509. 10846192 10.1136/jech.54.7.502 PMC1731716

[3] Dew K , Keefe V , Small K . 'Choosing' to work when sick: workplace presenteeism. Soc Sci Med. 2005;60(10):2273‐2282. 15748675 10.1016/j.socscimed.2004.10.022

[4] Turpin RS , Ozminkowski RJ , Sharda CE , et al. Reliability and validity of the Stanford Presenteeism Scale. J Occup Environ Med. 2004;46(11):1123‐1133. 15534499 10.1097/01.jom.0000144999.35675.a0

[5] Goetzel RZ , Long SR , Ozminkowski RJ , et al. Health, absence, disability, and presenteeism cost estimates of certain physical and mental health conditions affecting U.S. employers. J Occup Environ Med. 2004;46(4):398‐412. 15076658 10.1097/01.jom.0000121151.40413.bd

[6] Hemp P . Presenteeism: at work–but out of it. Harvard Bus Rev. 2004;82(10):49‐58. 15559575

[7] Miraglia M , Johns G . Going to work ill: a meta‐analysis of the correlates of presenteeism and a dual‐path model. J Occup Health Psychol. 2016;21(3):261‐283. 26550958 10.1037/ocp0000015

[8] Skagen K , Collins AM . The consequences of sickness presenteeism on health and wellbeing over time: a systematic review. Soc Sci Med. 2016;161:169‐177. 27310723 10.1016/j.socscimed.2016.06.005

[9] Gustafsson K , Marklund S . Consequences of sickness presence and sickness absence on health and work ability: a Swedish prospective cohort study. Int J Occup Med Environ Health. 2011;24(2):153‐165. 21526385 10.2478/s13382-011-0013-3

[10] Schultz IZ , Crook J , Berkowitz J , Milner R , Meloche GR . Predicting return to work after low back injury using the Psychosocial Risk for Occupational Disability Instrument: a validation study. J Occup Rehabil. 2005;15(3):365‐376. 16119227 10.1007/s10926-005-5943-9

[11] Kivimäki M , Head J , Ferrie JE , et al. Working while ill as a risk factor for serious coronary events: the Whitehall II study. Am J Public Health. 2005;95(1):98‐102. 15623867 10.2105/AJPH.2003.035873 PMC1449859

[12] Bergstrom G , Bodin L , Hagberg J , Aronsson G , Josephson M . Sickness presenteeism today, sickness absenteeism tomorrow? a prospective study on sickness presenteeism and future sickness absenteeism. J Occup Environ Med. 2009;51(6):629‐638. 19448572 10.1097/JOM.0b013e3181a8281b

[13] Bergstrom G , Hagberg J , Busch H , Jensen I , Bjorklund C . Prediction of sickness absenteeism, disability pension and sickness presenteeism among employees with back pain. J Occup Rehabil. 2014;24(2):278‐286. 23771777 10.1007/s10926-013-9454-9 PMC4000420

[14] Collins JJ , Baase CM , Sharda CE , et al. The assessment of chronic health conditions on work performance, absence, and total economic impact for employers. J Occup Environ Med. 2005;47(6):547‐557. 15951714 10.1097/01.jom.0000166864.58664.29

[15] Niven K , Ciborowska N . The hidden dangers of attending work while unwell: A survey study of presenteeism among pharmacists. Int J Stress Manage. 2015;22:207‐221.

[16] McKevitt C , Morgan M . Illness doesn't belong to us. J R Soc Med. 1997;90(9):491‐495. 9370984 10.1177/014107689709000907 PMC1296526

[17] Rosvold EO , Bjertness E . Physicians who do not take sick leave: hazardous heroes? Scand J Public Health. 2001;29(1):71‐75. 11355720

[18] Pilette PC . Presenteeism in nursing: a clear and present danger to productivity. J Nurs Adm. 2005;35(6):300‐303. 15951705 10.1097/00005110-200506000-00006

[19] Shamansky SL . Presenteeism…or when being there is not being there. Public Health Nurs. 2002;19(2):79‐80. 11860591 10.1046/j.1525-1446.2002.19201.x

[20] Crout LA , Chang E , Cioffi J . Why do registered nurses work when ill? J Nurs Adm. 2005;35(1):23‐28. 15647665 10.1097/00005110-200501000-00010

[21] Brborovic H , Daka Q , Dakaj K , Brborovic O . Antecedents and associations of sickness presenteeism and sickness absenteeism in nurses: a systematic review. Int J Nurs Pract. 2017;23(6):e12598. 10.1111/ijn.12598 29094426

[22] Demerouti E , Le Blanc PM , Bakker AB , Schaufeli WB , Hox J . Present but sick: A three‐wave study on job demands, presenteeism and burnout. Career Dev Int. 2009;14(50):50‐68.

[23] Rantanen I , Tuominen R . Relative magnitude of presenteeism and absenteeism and work‐related factors affecting them among health care professionals. Int Arch Occup Environ Health. 2011;84(2):225‐230. 21140162 10.1007/s00420-010-0604-5

[24] Elstad JI , Vabo M . Job stress, sickness absence and sickness presenteeism in Nordic elderly care. Scand J Public Health. 2008;36(5):467‐474. 18635730 10.1177/1403494808089557

[25] Gustafsson Senden M , Schenck‐Gustafsson K , Fridner A . Gender differences in Reasons for Sickness Presenteeism—a study among GPs in a Swedish health care organization. Ann Occup Environ Med. 2016;20:50. 10.1186/s40557-016-0136-x PMC5028976 27660717

[26] Umann J , Guido Lde A , Grazziano ES . Presenteeism in hospital nurses. Rev Lat Am Enfermagem. 2012;20(1):159‐166. 22481734 10.1590/s0104-11692012000100021

[27] Dhaini S , Zúñiga F , Ausserhofer D , et al. Absenteeism and presenteeism among care workers in swiss nursing homes and their association with psychosocial work environment: a multi‐site cross‐sectional study. Gerontology. 2015;62(4):386‐395. 26618789 10.1159/000442088

[28] SWEA . Arbetsmiljön 2015. [The Work Environment 2015]. Stockholm, Sweden: Arbetsmiljöverket [Swedish Work Environment Authority]; 2016.

[29] Conway PM , Hogh A , Rugulies R , Hansen AM . Is sickness presenteeism a risk factor for depression? a Danish 2‐year follow‐up study. J Occup Environ Med. 2014;56(6):595‐603. 24854252 10.1097/JOM.0000000000000177

[30] Campo M , Darragh AR . Work‐related musculoskeletal disorders are associated with impaired presenteeism in allied health care professionals. J Occup Environ Med. 2012;54(1):64‐70. 22157700 10.1097/JOM.0b013e31823c768a

[31] Bergstrom G , Bodin L , Hagberg J , Lindh T , Aronsson G , Josephson M . Does sickness presenteeism have an impact on future general health? Int Arch Occup Environ Health. 2009;82(10):1179‐1190. 19504117 10.1007/s00420-009-0433-6

[32] Dellve L , Hadzibajramovic E , Ahlborg G Jr . Work attendance among healthcare workers: prevalence, incentives, and long‐term consequences for health and performance. J Adv Nurs. 2011;67(9):1918‐1929. 21466574 10.1111/j.1365-2648.2011.05630.x

[33] SIA . Social Insurance in Figures 2013. Stockholm, Sweden: Swedish Social Insurance Agency (SIA); 2013.

[34] Hansen CD , Andersen JH . Going ill to work–what personal circumstances, attitudes and work‐related factors are associated with sickness presenteeism? Soc Sci Med. 2008;67(6):956‐964. 18571821 10.1016/j.socscimed.2008.05.022

[35] Lidwall U , Bergendorff S , Voss M , Marklund S . Long‐term sickness absence: changes in risk factors and the population at risk. Int J Occup Med Environ Health. 2009;22(2):157‐168. 19617194 10.2478/v10001-009-0018-3

[36] Krane L , Larsen EL , Nielsen CV , Stapelfeldt CM , Johnsen R , Risor MB . Attitudes towards sickness absence and sickness presenteeism in health and care sectors in Norway and Denmark: a qualitative study. BMC Public Health. 2014;14:880‐893. 25160059 10.1186/1471-2458-14-880 PMC4168251

[37] Chambers C , Frampton C , Barclay M . Presenteeism in the New Zealand senior medical workforce—a mixed‐methods analysis. Age. 2017;130(1449):10‐21. 28178725

[38] Hansen CD , Lund T , Labriola M . Is it masculine to turn up ill at work? Eur J Pub Health. 2011;21(Suppl):1.21247865

[39] Martinez LF , Ferreira AI . Sick at work: presenteeism among nurses in a Portuguese public hospital. Stress Health. 2012;28(4):297‐304. 22282226 10.1002/smi.1432

[40] Gerich J . Sickness presence, sick leave and adjustment latitude. Int J Occup Med Environ Health. 2014;27(5):736‐746. 25257339 10.2478/s13382-014-0311-7

